# Sudden onset pacemaker-induced diaphragmatic twitching during general anesthesia

**DOI:** 10.1186/s40981-019-0257-7

**Published:** 2019-06-03

**Authors:** Kanae Tanabe, Masakazu Kotoda, Daiki Nakashige, Kazuha Mitsui, Kodai Ikemoto, Takashi Matsukawa

**Affiliations:** Department of Anesthesiology, University of Yamanashi, 1110 Shimokato, Chuo, Yamanashi 409-3898 Japan

**Keywords:** Atrial pacing, Diaphragmatic twitching, General anesthesia, Pacemaker, Phrenic nerve

## Abstract

**Background:**

Involuntary muscle contraction caused by extracardiac stimulation is a rare complication induced by a pacemaker. We report a case who developed sudden onset diaphragmatic contractions during general anesthesia caused by a DDD mode pacemaker.

**Case presentation:**

A 74-year-old woman with a permanent pacemaker was scheduled to undergo mastectomy. The pacing mode was switched from DDD to VOO intraoperatively to avoid electromagnetic interference. Immediately after returning the pacing mode to DDD after surgery, diaphragmatic contractions occurred, mimicking bucking type of movements. After switching the pacing to A-sense V-pace, the twitching ceased. Because no structural problems were noted, and the twitching disappeared after terminating atrial pacing, diaphragmatic contractions might be caused by stimulation of the right phrenic nerve located near the right appendage where the electrode was installed.

**Conclusion:**

The potential risk of muscle twitching should be carefully evaluated preoperatively especially in patients with atypical position of pacemaker leads.

## Background

Pacemaker-induced extracardiac stimulation causes involuntary muscle twitching, which involves the pectoral and intercostal muscles and the diaphragm [1–3]. We herein report a rare case with a dual-chamber pacemaker who developed sudden onset diaphragmatic contractions during general anesthesia.

## Case presentation

A 74-year-old woman (height, 146 cm; weight, 36 kg) with cancer of the right breast was scheduled to undergo mastectomy under general anesthesia. The patient’s medical history included permanent dual-chamber pacemaker implantation (Adapta L ADDRL1, Medtronic, Dublin, Ireland) for complete atrioventricular block at the age of 59. The pacemaker had been programmed in DDD mode at 60–120 beats/min with atrial output 1.5 V-0.4 ms, ventricular output 1.5 V-0.4 ms, atrial sensitivity 0.5 mV, and ventricular sensitivity 2.8 mV. The patient’s preoperative electrocardiogram showed A-sense V-pace with an intrinsic atrial rate of 84 beats/min.

In the operating room, general anesthesia was induced with fentanyl 100 μg, remifentanil 0.2 μg/kg/min, propofol 50 mg, and rocuronium 40 mg intravenously. After tracheal intubation, the pacemaker mode was switched to VVI mode. The patient was pacemaker dependent, and her intrinsic ventricular rate was less than 40 beats/min. The ventricular pacing threshold (0.6 V-0.4 ms) was confirmed. The pacing mode was changed to VOO at a fixed rate of 80 beats/min intraoperatively because the surgical site was very close to the generator, and we expected electromagnetic interference due to the use of unipolar electrocautery. General anesthesia was maintained with 3.5% desflurane and 0.2–0.4 μg/kg/min of remifentanil. The patient remained hemodynamically stable intraoperatively, and the surgical procedure was uneventful.

**Fig. 1:**
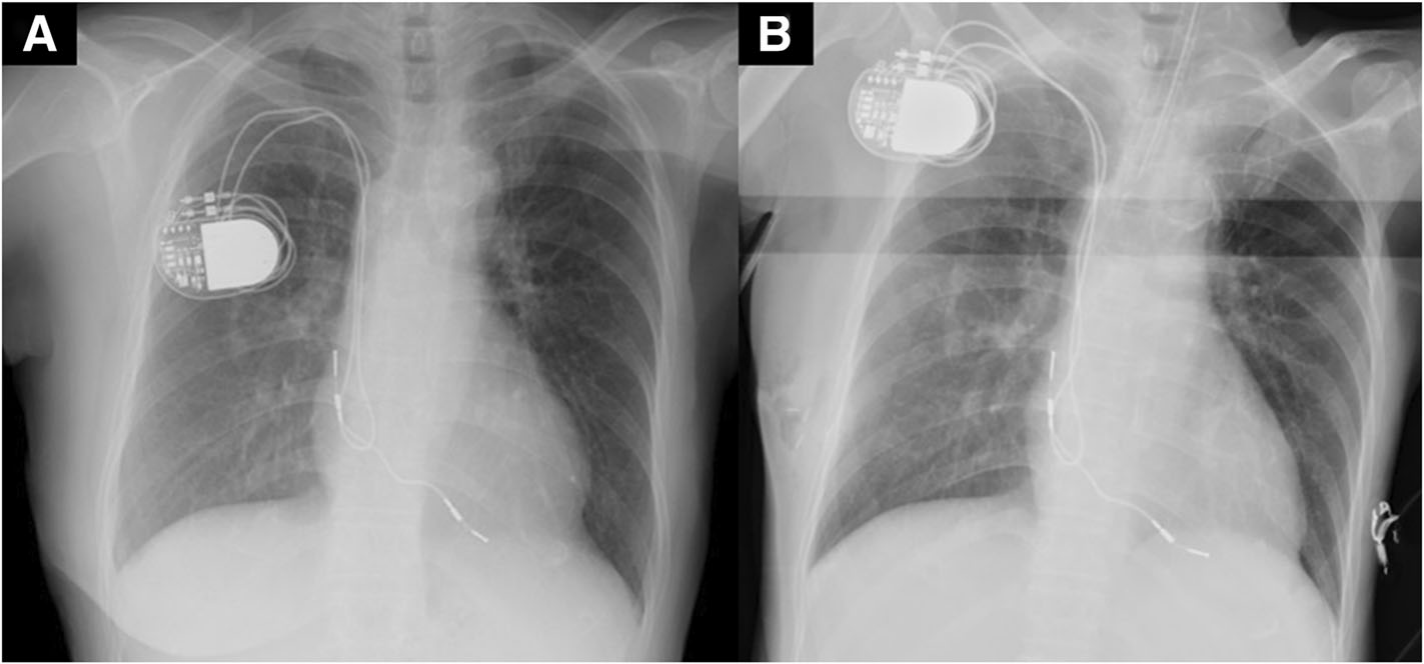
Preoperative (**a**) and postoperative (**b**) chest radiograph**.** Although the postoperative chest radiograph showed a slight change in generator position compared to the preoperative chest radiograph, no structural problems, including displacement of the pacing leads, perforation, cardiac dilatation, or pericardial effusion, were noted

Prior to recovery from general anesthesia, the pacemaker was programmed to the preoperative setting, in DDD mode at a pacing rate of 60–120 beats/min. Immediately afterwards, diaphragmatic twitching was observed, mimicking bucking type of movements. The electrocardiogram of the patient showed AV sequential pacing; after the minimum heart rate was set to 40 beats/min, the pacing switched to A-sense V-pace, and the twitching ceased. The twitching was reproducible by arterial pacing with a threshold of 1.5 V-0.4 ms, same as the original atrial output. The patient’s intrinsic atrial rate was approximately 50 beats/min. No structural problems, including displacement of the pacing leads, perforation, cardiac dilatation, or pericardial effusion were noted on the chest radiograph (Fig. 1). The atrial pacing threshold (0.6 V-0.4 ms) and impedance (611 Ω) was not modified after a generator change at the age of 68. After recovery from general anesthesia and extubation, the patient’s intrinsic atrial rate increased to 80–90 beats/min. The DDD pacing range was switched to the preoperative setting (60–120 beats/min), and the patient was transferred to the ward. The postoperative course was uneventful, and no further diaphragmatic twitching was observed. The patient was discharged on postoperative day 4.

## Discussion

**Fig. 2:**
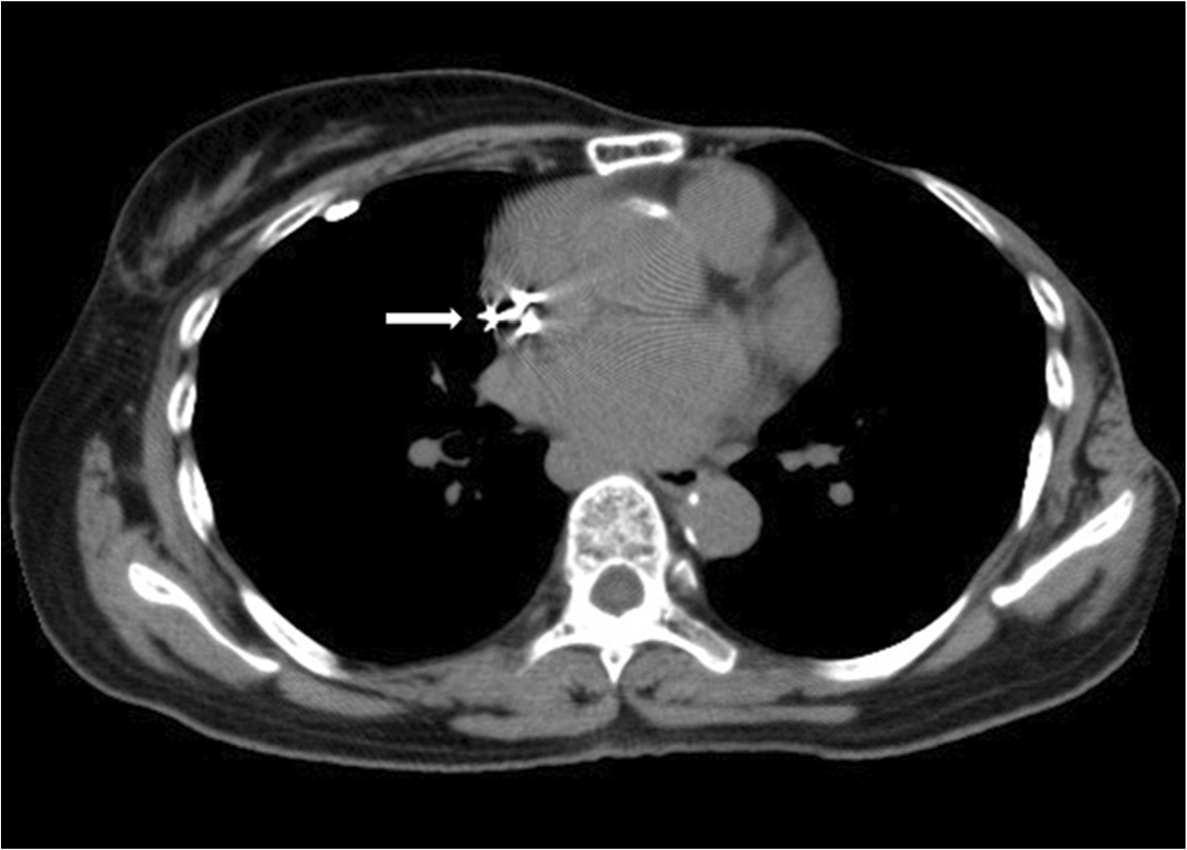
Preoperative chest computed tomography**.** The atrial lead tip (arrow) was placed in the high lateral part of the right atrium

Muscle twitching is one of the complications caused by a permanent cardiac pacemaker, and Twiddler syndrome, where twisting or rotating of the generator in its pocket results in lead rotation or coiling, is a common cause of this [4]. Other causes of pacemaker-induced twitching include spontaneous migration of pacemaker leads or generator, cardiac perforation, electric current leakage, and inappropriate output [5–7]. Therefore, the position, morphology, and settings must be checked when unexpected muscle twitching occurs in a patient with an implanted pacemaker. In the present case, atrial, but not ventricular, pacing caused extracardiac twitching. Although the postoperative chest radiograph showed a slight change in generator position, the pacing leads were not displaced. In addition, the pacemaker threshold and impedance were normal, indicating that electrical leakage or disconnection of leads did not lead to muscle twitching. The preoperative computed tomography of our patient revealed that the atrial lead tip was placed in the high lateral part of the right atrium (Fig. 2). Because the right phrenic nerve courses near the lateral wall of the right atrium, stimulation of the right phrenic nerve would have been the cause of diaphragmatic twitching, as reported by Khan et al. [3]. In retrospect, we discovered that the patient had occasionally noticed rhythmic contractions of the diaphragm at night. The patient’s intrinsic atrial rate may have decreased below 60 beats/min during sleep, triggering atrial pacing and phrenic nerve stimulation. However, because the pacemaker lead in this patient was implanted 15 years ago, the position of the atrial lead could not be changed due to the possible risk of cardiac perforation. In addition, the atrial output could not be lowered below 1.5 V to avoid pacing failure. Against these backgrounds, a decrease in the intrinsic atrial rate followed by triggering of atrial pacing probably led to unexpected diaphragmatic twitching during general anesthesia in our patient.

In the present case, VOO mode was selected to avoid electromagnetic interference due to the proximity of the generator to the surgical site. DDD mode is widely used intraoperatively if the surgical site is not close to the generator [8] and considered to be superior to VOO as it maintains the “atrial kick” and sequential atrioventricular contraction [9]. If DDD mode is selected, caution should be exercised because muscle twitching can be induced by atrial pacing as occurred in this case. In patients with a dual-chamber pacemaker, atrial pacing should be tested to evaluate thresholds and the presence of twitching. If twitching is induced by atrial pacing, reprogramming of the pacemaker with appropriate anesthetic and hemodynamic management is required. The atrial rate may need to be maintained higher than the pacing range; alternatively, the lower limit of the pacing rate should be reduced during surgery to avoid sudden muscle twitching.

Sudden, unexpected bucking movement during general anesthesia can be hazardous. Anesthesiologists should be aware of the possibility of extracardiac stimulation especially in a patient with atypical positioning of the pacemaker leads. A careful, detailed preoperative evaluation, including meticulous interview of the patient regarding any symptoms related to the pacemaker, should be performed. Although the details about the pacemaker and information on the time of implantation may not always be available, every effort should be made to obtain such information to avoid sudden muscle twitching [10].

In summary, we describe a patient with a permanent pacemaker implanted for complete atrioventricular block who presented with sudden bucking-like diaphragmatic contraction during general anesthesia. The potential risk of muscle twitching should be carefully evaluated preoperatively especially in patients with atypical position of pacemaker leads. We recommend testing of atrial pacing prior to surgery in these patients.

## Data Availability

Not applicable.
